# Differentially Evolved Genes of *Salmonella* Pathogenicity Islands: Insights into the Mechanism of Host Specificity in *Salmonella*

**DOI:** 10.1371/journal.pone.0003829

**Published:** 2008-12-03

**Authors:** Sandeepa M. Eswarappa, Jessin Janice, Arvindhan G. Nagarajan, Sudhagar V. Balasundaram, Guruswamy Karnam, Narendra M. Dixit, Dipshikha Chakravortty

**Affiliations:** 1 Department of Microbiology and Cell Biology, Centre for Infectious Disease Research and Biosafety Laboratories, Indian Institute of Science, Bangalore, India; 2 Department of Chemical Engineering, Centre for Infectious Disease Research and Biosafety Laboratories, Indian Institute of Science, Bangalore, India; Centre for DNA Fingerprinting and Diagnostics, India

## Abstract

**Background:**

The species *Salmonella enterica* (*S*. *enterica*) includes many serovars that cause disease in avian and mammalian hosts. These serovars differ greatly in their host range and their degree of host adaptation. The host specificity of *S*. *enterica* serovars appears to be a complex phenomenon governed by multiple factors acting at different stages of the infection process, which makes identification of the cause/s of host specificity solely by experimental methods difficult.

**Methodology/Principal Findings:**

In this study, we have employed a molecular evolution and phylogenetics based approach to identify genes that might play important roles in conferring host specificity to different serovars of *S*. *enterica*. These genes are ‘differentially evolved’ in different *S*. *enterica* serovars. This list of ‘differentially evolved’ genes includes genes that encode translocon proteins (SipD, SseC and SseD) of both *Salmonella* pathogenicity islands 1 and 2 encoded type three secretion systems, *sptP*, which encodes an effector protein that inhibits the mitogen-activated protein kinase pathway of the host cell, and genes which encode effector proteins (SseF and SifA) that are important in placing the *Salmonella*-containing vacuole in a juxtanuclear position.

**Conclusions/Significance:**

Analysis of known functions of these ‘differentially evolved genes’ indicates that the products of these genes directly interact with the host cell and manipulate its functions and thereby confer host specificity, at least in part, to different serovars of *S*. *enterica* that are considered in this study.

## Introduction

The genus *Salmonella* comprises Gram-negative bacteria and includes two species, *Salmonella bongori* (*S. bongori*) *and Salmonella enterica* (*S. enterica*) [1]. The lineage of *S. enterica* is thought to have branched into several distinct phylogenetic groups or subspecies. *S. enterica* subspecies I is most frequently isolated from avian and mammalian hosts while *S. bongori* and *S. enterica* subspecies II, IIIa, IIIb, IV, VI, and VII are mainly associated with cold-blooded vertebrates [2]. *S. enterica* subspecies are further classified into more than 2000 serovars, which include pathogens having great medical and veterinary importance. These serovars differ greatly in their host range and their degree of host adaptation [2]. For example, *Salmonella enterica* serovar Dublin (*S*. Dublin) infects cattle; *Salmonella enterica* serovar Choleraesuis (*S*. Choleraesuis) infects pigs and other mammals; *Salmonella enterica* serovar Gallinarum (*S*. Gallinarum) infects poultry; *Salmonella enterica* serovar Typhimurium (*S*. Typhimurium) and *Salmonella enterica* serovar Enteritidis (*S*. Enteritidis) infect multiple hosts including humans, rodents, cattle, poultry and sheep; *Salmonella enterica* serovar Typhi (*S*. Typhi) and *Salmonella enterica* serovar Paratyphi (*S*. Paratyphi) infect humans. In humans, the extent of disease caused by different serovars of *S*. *enterica* varies from mild enteritis to the life threatening typhoid fever. No other known bacterial pathogens belonging to a single species show such a remarkable variation in their host specificity. Yet, the close DNA relatedness of the *S*. *enterica* serovars suggests that they are clonal in their origin [2].

Several experimental studies have already been attempted to unravel the mechanistic origins of the host specificity of *S*. *enterica* serovars [3]–[7]. For instance, it has been shown that the host specificity of *S*. *enterica* serovars in sheep is not related to its ability to invade the intestinal epithelium [3]. The avirulence of *S*. Gallinarum in mice, however, is due to its inability to enter the intestinal epithelium, whereas in calves it is due to its inability to disseminate from mesenteric lymph nodes [5], [6]. Another experimental study reports that the host specificity of *S*. *enterica* in chicken and mice primarily occurs at the level of the reticuloendothelial system [4]. Host specificity in *S*. *enterica* serovars thus appears to be a complex phenotype imparted by multiple genes functioning at different stages of infection and cannot be attributed to a single virulence determinant. It is proposed that the genes belonging to *Salmonella* pathogenicity islands, virulence plasmid, fimbrial operons, pseudogenes and lysogenic phages are important in conferring host specificity and restricting the host range [2], [8]. The large number of genes involved perhaps underlies the failure of attempts to extend the host range of host-restricted *Salmonella* by transfer of small segments of a broad host-range serovar genome [9] and also renders experimental elucidation of the mechanisms underlying host specificity difficult. Nevertheless, *Salmonella* serves as a good model system to understand the phenomenon of host adaptation by bacterial pathogens as the virulence factors of *Salmonella* are well characterized. Elucidation of host adaptation mechanisms is expected to have broad implications for understanding the emergence of new pathogens and for vaccine design.

*Salmonella,* like many pathogenic bacteria, harbors clusters of virulence genes that are acquired by horizontal gene transfer; the evolution of virulence in *Salmonella* is driven by such horizontal gene transfer. These gene clusters, termed *Salmonella* pathogenicity islands (SPIs), are considered to be ‘quantum leaps’ in bacterial evolution [10]. SPI-1 is located at 63 min in the *S.* Typhimurium genome and is a 40 kb island with a major role in the invasion of host cells [11], [12]. SPI-2 is located at 31 min and is also a 40 kb island that confers the ability to survive within host cells, especially macrophages [13], [14]. Both SPI-1 and SPI-2 encode different type three secretory systems (TTSS). SPI-3 is located at 82 min, is 17 kb long, and plays a role in intra-macrophage survival and virulence [15]. SPI-4 is located at 92 min, is 27 kb long, and is implicated in the adhesion of *Salmonella* to host epithelial cells [16]. SPI-5 is located at 20 min and is required for enteropathogenicity [17]. SPI genes thus encode many virulence factors that are involved directly in manipulating the host system and may be responsible, at least in part, for the host specificity of different *S*. *enterica* serovars.

We hypothesized that the genes that confer host specificity in *S*. *enterica* must have evolved differentially in different serovars in response to the differential influences of their specific hosts. Our aim in this study was to identify SPI genes that are differentially evolved in different *S*. *enterica* serovars. We have chosen *S.* Typhi (Ty2), *S.* Paratyphi A, *S.* Typhimurium, *S.* Enteritidis and *S.* Choleraesuis for our study. Using a molecular evolution and phylogenetics based approach, we identified six genes as ‘differentially evolved genes’. Analysis of putative/proven function/s of these differentially evolved genes provides insights into the complex phenomenon of host specificity in *S*. *enterica*.

## Results and Discussion

### Identification of differentially evolved genes

In this study, we have analyzed genes belonging to SPI-1 (39 genes), SPI-2 (38 genes), SPI-3 (6 genes), SPI-4 (5 genes) and SPI-5 (7 genes) of *S.* Typhi (Ty2), *S.* Paratyphi A, *S.* Typhimurium, *S.* Choleraesuis, and *S.* Enteritidis (Table S1, S2, S3, S4 and S5). We have also considered genes located outside SPIs that encode proteins secreted through either SPI-1 or SPI-2 encoded TTSS. Pseudo genes and genes that did not have homologues in all of the serovars considered were excluded from our analysis (Table S6). *S*. Gallinarum was very closely related to *S*. Enteritidis with respect to all the above SPI genes (data not shown). Consequently, inclusion of *S*. Gallinarum in our analysis did not alter the results significantly.

### Analysis based on non-synonymous distance

To identify differentially evolved genes, we determined the non-synonymous distances (D_N_) between the homologues of individual SPI genes in all possible pairs of the above serovars. D_N_ is a measure of the degree to which two homologous coding sequences differ in their amino acid content. Specifically, it indicates the degree to which two sequences differ at non-synonymous sites, i.e., nucleotide sites at which a substitution causes an amino acid change. Differentially evolved genes are thus expected to have relatively large values of D_N_ in one or more serovar combinations. We therefore examined the maximum value of D_N_ for each gene (out of ten D_N_ values corresponding to ^5^C_2_ = 10 serovar combinations). We found that nine and twenty eight genes had a maximum D_N_ value (D_N_^max^) of greater than 0.02 and 0.01 (data not shown) respectively. *sipD*, *sptP*, *prgI*, *sseC*, *sseD*, *sseF*, *ssaI, sifA* and STM1089 are the nine potential ‘differentially evolved genes’ whose D_N_^max^ values were greater than 0.02.

### Phylogenetic analysis

To establish the differentially evolved genes, we next compared the phylogeny of the nine potential ‘differentially evolved genes’ identified above (with D_N_^max^>0.02) with the phylogeny of the *S*. *enetrica* species and with the phylogeny of the five pathogenecity islands of *Salmonella* (SPI-1 to SPI-5). We inferred the species phylogeny from *dnaB* and *16S rRNA*, two house keeping genes. The phylogeny of the five pathogenecity islands was inferred from the consensus tree of 95 trees based on 95 SPI genes. Analysis of maximum likelihood (ML) trees of the 95 SPI genes revealed that the phylogeny of *ssaS*, which encodes a protein that is a part of the SPI-2 encoded TTSS apparatus, represents the best tree. (The TREE-PUZZLE 5.2 program was used for all these analysis [18]).

We employed the Shimodaira-Hasegawa (SH) test [19] to verify whether the phylogenies of the nine potential ‘differentially evolved genes’ were significantly different from those of *dnaB* and *16S rRNA* (representing species phylogeny), and from those of the consensus tree and *ssaS* (representing the phylogeny of pathogenicity islands). A summary of the results of this analysis is presented in Table 1. The phylogenies of six out of the nine genes were significantly (*P*<0.05) different from those of *dnaB*, *16S rRNA, ssaS* and the consensus tree. *prgI*, *ssaI* and STM1089 failed this test (*P*>0.05). This analysis demonstarted that the evolution of *sipD*, *sptP*, *sseC*, *sseD*, *sseF* and *sifA* is different from the rest of the genome including the five pathogenecity islands. Hence, we termed these six genes as ‘differentially evolved genes’ of SPI-1 to SPI-5 (Table 2).

**Table 1:** Summary of Shimodaira-Hasegawa tests

Data set		*vs. dnaB*	*vs. 16S rRNA*	*vs. ssaS*	*vs. consensus tree*
*sipD*	***sipD***	*P<*0.0001	*P<*0.0001	*P<*0.0001	*P<*0.0001
*sptP*	***sptP***	*P<*0.0001	*P<*0.0001	*P<*0.0001	*P<*0.0001
*prgI*	***prgI*** *	*P = *0.0670	*P = *0.0650	*P = *0.0630	*P* = 0.057
*sseC*	***sseC***	*P<*0.0001	*P<*0.0001	*P<*0.0001	*P<*0.0001
*sseD*	***sseD***	*P<*0.0001	*P<*0.0001	*P<*0.0001	*P<*0.01
*sseF*	***sseF***	*P<*0.0001	*P<*0.05	*P<*0.0001	*P<*0.0001
*ssaI*	***ssaI*** *	*P = *0.4320	*P = *0.4280	*P = *0.5620	*P* = 0.3050
*sifA*	***sifA***	*P<*0.0001	*P<*0.0001	*P<*0.0001	*P<*0.0001
*STM1089*	***STM1089*** *	*P = *0.0660	*P = *0.1470	*P = *0.0670	*P* = 0.063

* These genes failed the SH test

**Table 2:** Differentially evolved genes of SPI-1 to SPI-5

Gene	SPI	D_N_^max^	Function
*sipD*	SPI-1	0.0733	Translocon component of SPI-1 encoded TTSS and is required for the secretion of other effector proteins [23], [24].
*sptP*	SPI-1	0.0295	Effector protein of SPI-1 encoded TTSS and is a protein tyrosine phosphatase [38].
*sseC*	SPI-2	0.0283	Translocon component of SPI-2 encoded TTSS and is required for the secretion of other effector proteins[34], [35].
*sseD*	SPI-2	0.0254	Translocon component of SPI-2 encoded TTSS and is required for the secretion of other effector proteins [34], [35].
*sseF*	SPI-2	0.0254	Effector protein secreted through SPI-2 encoded TTSS; involved in positioning of SCV by recruiting dynein [49].
*sifA*	-	0.0321	Effector protein secreted through SPI-2 encoded TTSS; involved in positioning of SCV [51], [52].

**Figure 1:**
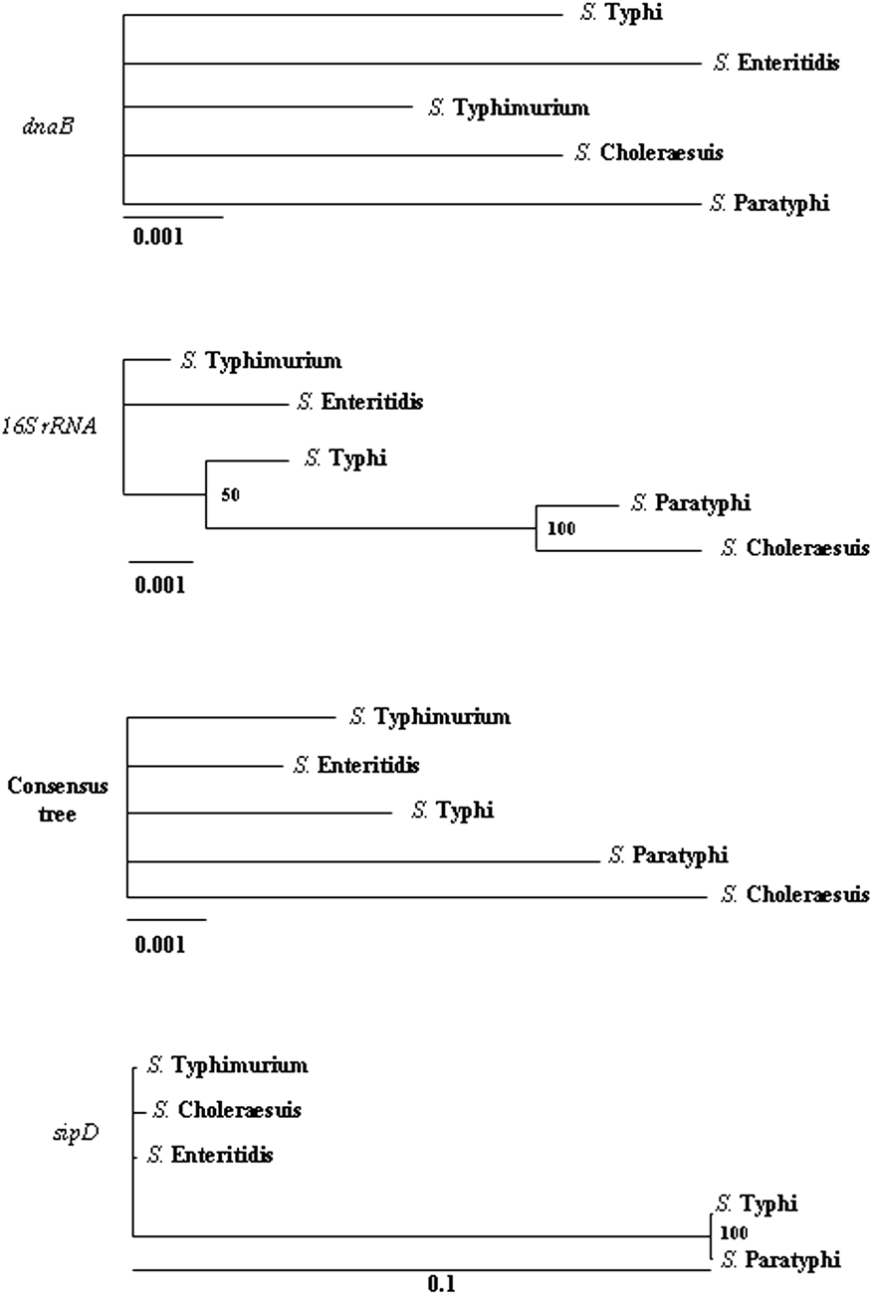
Molecular phylogeny of *S. enterica* serovars. ML trees constructed based on *dnaB*, *16S rRNA*, *sipD* and the consensus tree of SPI-1 to SPI-5 genes. Numbers inside the trees represent support values for the internal branches. Scale bars represent ML distance.

**Figure 2:**
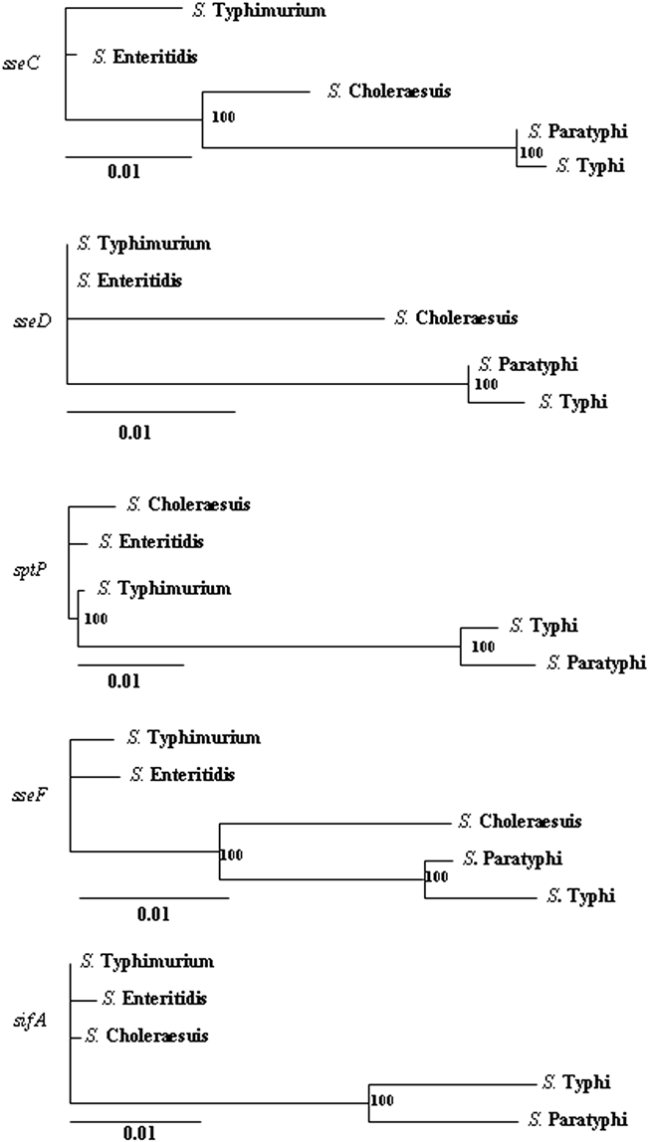
Molecular phylogeny of *S. enterica* serovars inferred from differentially evolved genes. ML trees constructed based on *sseC*, *sseD*, *sptP*, *sseF* and *sifA*. Numbers inside the trees represent support values for the internal branches. Scale bars represent ML distance.

In ML trees based on *dnaB, 16S rRNA, ssaS* (data not shown) and the consensus tree, serovars did not cluster according to their host specificity. However, in ML trees based on differentially evolved genes, human adapted serovars (*S*. Typhi and *S*. Paratyphi) clustered together (Fig. 1 and 2). Further statistical analysis confirmed that these genes have evolved differentially in different *S*. *enterica* serovars according to their host specificity (Table 3). Next, we examined, whether the evolution of these genes is significantly accelerated in any particular serovar. We compared the branch lengths of each serovar obtained from ML trees based on six differentially evolved genes. Interestingly, we found that the branch lengths of *S*. Typhi and *S*. Paratyphi are significantly larger (4 to 30 fold) than those of other serovars (Table 4). The evolution of the differentially evolved genes thus appears to be accelerated in human specific serovars suggesting a role for these genes in the host adaptation of human specific serovars.

**Table 3:** Comparison of D_N_ values of *S*. *enterica* serovar combinations with similar and dissimilar host specificity*.

	(STM-SEN and STY-SPA) vs. (STY-STM, STY-SEN, SPA-STM and SPA-SEN)†
***sipD***	*P*<0.00001
***sseC***	*P*<0.001
***sseD***	*P*<0.00001
***sptP***	*P*<0.0001
***sseF***	*P*<0.05
***sifA***	*P*<0.05
***prgI*** #	*P* = 0.12
***ssaI*** #	*P* = 1
***ssaS*** #	*P* = 1

* Student's t-test was performed to calculate the *P* values.

† STM- *S.* Typhimurium; SEN- *S.* Enteritidis; STY- *S.* Typhi; SPA- *S.* Paratyphi.

# Genes that are not differentially evolved failed this test.

**Table 4:** Analysis of maximum likelihood branch lengths of different serovars obtained from ML trees based on the differentially evolved genes

	*sipD*	*sseC*	*sseD*	*sptP*	*sseF*	*sifA*	(Mean±SE)
***S*** **. Typhi ** *	0.10075	0.03843	0.02761	0.04038	0.03097	0.03617	0.0457±0.0112
***S*** **. Typhimurium**	0.00097	0.00948	0.00001	0.00186	0.00301	0.00001	0.00256±0.00146
***S*** **. Paratyphi ** *	0.10075	0.03637	0.02421	0.04355	0.02544	0.03457	0.0441±0.0117
***S*** **. Choleraesuis**	0.00194	0.01978	0.01895	0.00432	0.02542	0.00099	0.0119±0.00436
***S*** **. Enteritidis**	0.00097	0.00091	0.00001	0.00183	0.00340	0.00198	0.00152±0.00048

* Branch lengths of *S*. Typhi and *S*. Paratyphi are significantly different from those of other serovars (*P*<0.05; Student's t-test), however the difference between themselves is not significant (*P* = 0.92; Student's t-test).

### Genes encoding the translocon proteins of SPI-1 and SPI-2 encoded TTSSs are differentially evolved

Serovars belonging to *S*. *enterica* possess two TTSS: one encoded in SPI-1 and the other one in SPI-2. The TTSS encoded in SPI-1 is required for the entry of *Salmonella* into the host epithelial cells [20], [21]. Entry into the host system is a potential determinant of host specificity [5]. The TTSS encoded in SPI-2 enables *S. enterica* to modify functions of the host cell, and thus is essential for the survival and replication of *S. enterica* inside host macrophages, which is vital for causing systemic infection [14]. Intracellular survival and replication is also a potential determinant of host specificity [22]. These TTSSs are used by *Salmonella* to inject effector proteins into the host cytoplasm by piercing the cell membrane or the vacuolar membrane. Thus, the translocons of the TTSS encoded in SPI-1 and SPI-2 interact directly with the host epithelial cell membrane and the vacuolar membrane, respectively. Therefore, these membranes are likely to have influenced the evolution of genes encoding the translocon proteins of the TTSSs. Indeed, our analysis revealed that *sipD*, which encodes a translocon protein of SPI-1 encoded TTSS, and *sseC* and *sseD* which encode translocon proteins of SPI-2 encoded TTSS, are differentially evolved.

### Differential evolution of *sipD*

SipD is a translocon protein of SPI-1 encoded TTSS and plays a vital role in the translocation of secreted proteins into host cells [23], [24]. *sipD* null mutants are non-invasive in cultured epithelial cells [23]. IpaD of *Shigella* and LcrV of Yersinia are homologues of SipD and are known to localize to the TTSS needle tip; the tip complex assists with the assembly of the translocation pore, serving as an assembly platform [25]–[27]. In our analysis, *sipD* had the maximum D_N_^max^ value among the differentially evolved genes, suggesting that *sipD* has evolved maximally differentially among the *S*. *enterica* serovars we considered (Table 2). We therefore examined this gene in detail.

**Figure 3:**
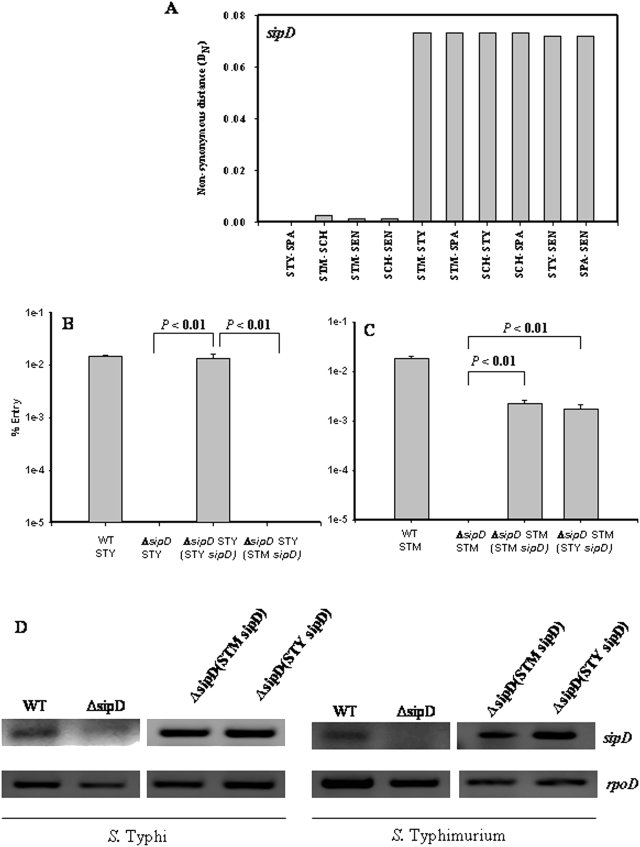
Differential evolution of *sipD*. (A) Non-synonymous distance profile of *sipD*. (B) Expression of SipD of *S.* Typhi but not SipD of *S.* Typhimurium enabled Δ*sipD S.* Typhi to enter HeLa cells. (C) Expression of SipD of either *S.* Typhi or *S.* Typhimurium enabled Δ*sipD S.* Typhimurium to enter HeLa cells. Graphs represent mean % entry and error bars represent standard error. Student's t-test was used to calculate the *P* value. (D) RT PCR analysis of expression of *sipD* in different strains of *S*. Typhi and *S*. Typhimurium. *rpoD* expression was used as internal control. STM- *S.* Typhimurium; STY- *S.* Typhi; SCH- *S.* Choleraesuis; SPA- *S.* Paratyphi; SEN- *S.* Enteritidis.

Remarkably, *sipD* showed zero D_N_ and synonymous distance (D_S_) values (data not shown) between *S.* Typhi and *S.* Paratyphi indicating that SipD is identical in human adapted serovars (Fig. 3A). SipD is also conserved among other serovars that are not well adapted to humans (D_N = _0.0013 to 0.0026). However, the D_N_ values of *sipD* between human adapted serovars (*S.* Typhi and *S.* Paratyphi) and other serovars (*S.* Typhimurium, *S.* Choleraesuis, and *S.* Enteritidis) were large (0.0719 to 0.0733) (Fig. 3A). Human adapted serovars thus appear to have evolved a SipD that is different from the SipD of other serovars.

### SipD of human adapted serovars is structurally different from that of other serovars

Alignment of predicted amino acid sequences of SipD revealed many disfavored amino acid changes between positions 180 and 280 and these changes were specific to human adapted serovars (Fig. S1; Materials and Methods S1). IpaD of *Shigella* shares 40% sequence identity with SipD [23]. In *Shigella*, central deletions in IpaD corresponding to amino acid positions 180 to 280 of SipD completely eliminate the invasion function of IpaD [28]. The region between amino acid positions 180 and 280 thus appears to be important for the function of SipD. Tertiary structure prediction revealed a prominent difference between the structure of the SipD of *S*. Typhimurium and that of *S*. Typhi in regions corresponding to the residues 47 to 57, 197 to 210 and 268 to 282 (Fig. S2; Materials and Methods S1).

### SipD of human adapted serovars is functionally different from that of *S.* Typhimurium

In order to verify whether the SipD of *S.* Typhi is functionally different from that of *S.* Typhimurium, we performed an invasion assay in HeLa cells. We observed that the wild type *S.* Typhi and *S.* Typhimurium could enter HeLa cells, but Δ*sipD S.* Typhi and Δ*sipD S.* Typhimurium, which lack *sipD*, could not. The entry defect of Δ*sipD S.* Typhi was abolished when the SipD of *S.* Typhi was expressed but not when the SipD of *S.* Typhimurium was expressed. However, the entry defect of Δ*sipD S.* Typhimurium was abolished when the SipD of either *S.* Typhi or *S.* Typhimurium was expressed (Fig. 3B and C). Similar results were obtained in Intestine 407 cells, a human intestine epithelial cell line (Fig. S3A and B). The SipD of *S.* Typhi is thus functionally different from that of *S.* Typhimurium. The expression of *sipD* in all the complimented strains was confirmed by RT PCR (Fig. 3D). Heterologous SipD expression did not affect the expression of SipC, another TTSS apparatus protein, which suggests that heterologous SipD expression does not interfere with the expression of the TTSS apparatus (Fig. S3C; Materials and Methods S1).

Typhoid fever, caused by *S.* Typhi, is characterized by a weak inflammatory response and punched out ulcers in the intestine, whereas gastroenteritis, caused by *S.* Typhimurium, is characterized by inflammatory changes involving neutrophil efflux and fluid accumulation without any ulcerations in the intestine [29], [30]. The early interactions of *S.* Typhi and *S.* Typhimurium with intestinal epithelial cells are different [31]. Moreover, *S.* Typhi, but not *S.* Typhimurium, uses cystic fibrosis transmembrane conductance regulator (CFTR) to enter human epithelial cells [32]. Thus, the invasion of human intestinal epithelium by *S.* Typhi is different from that of *S.* Typhimurium. Our analysis suggests that human adapted serovars have evolved a different SPI-1 encoded TTSS needle substructure, made up of a unique SipD that contributes to the ability of the human adapted serovars to colonize the human intestine differently from and perhaps more efficiently than other serovars that cause gastroenteritis. Identification of host proteins that interact with SipD will help understand the precise role of SipD in conferring human specificity to human adapted serovars of *S*. *enterica*.

Though the main contribution of SPI-1 to *Salmonella* pathogenesis is limited to the gastrointestinal phase of the disease, it has been shown recently that SipB, SipC and SipD of SPI-1 have a previously unappreciated role in the long-term systemic infection in mice [33]. It is possible that the SipD of human adapted serovars might play an important role in causing chronic infection and, possibly a carrier state in humans, which is common in typhoid fever caused by human adapted serovars but not in gastroenteritis caused by other serovars like *S.* Typhimurium.

### Differential evolution of *sseC* and *sseD*

SseC and SseD along with SseB form the translocon of SPI-2 encoded TTSS. Because of this vital function, SseC and SseD are required for the proliferation of *S. enterica* inside host cells and thus are essential for the virulence of *S. enterica* [34], [35]. SseD has limited sequence similarity to EspB of enteropathogenic *Escherichia coli*, whereas SseC is a member of the YopB family of translocon proteins involved in pore formation in the target membrane [36].

**Figure 4:**
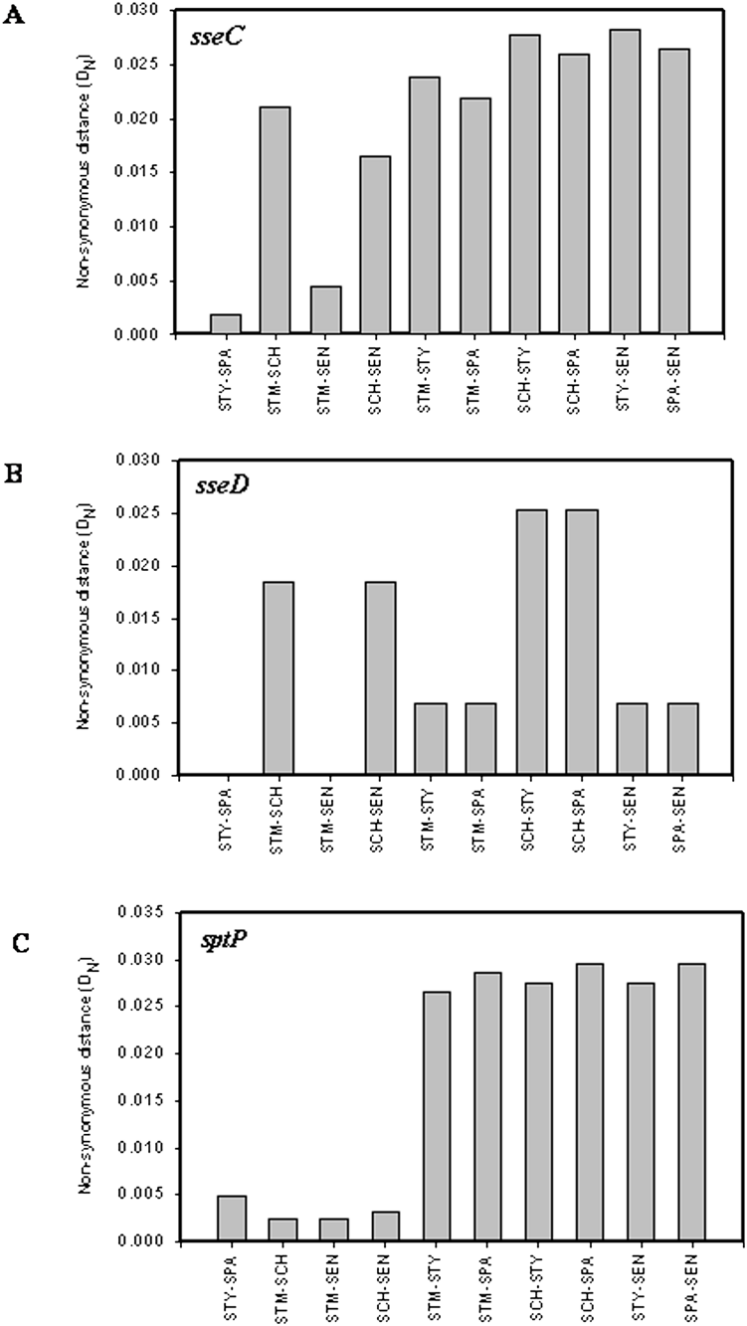
Differential evolution of *sseC*, *sseD* and *sptP*. (A) Non-synonymous distance profile of *sseC*. (B) Non-synonymous distance profile of *sseD*. (C) Non-synonymous distance profile of *sptP*. STM- *S.* Typhimurium; STY- *S.* Typhi; SCH- *S.* Choleraesuis; SPA- *S.* Paratyphi; SEN- *S.* Enteritidis.

D_N_ values of *sseC* between *S.* Typhi and *S.* Paratyphi and between *S.* Typhimurium and *S.* Enteritidis (0.0018 and 0.0044 respectively) were significantly smaller than the other combinations of serovars (0.0165 to 0.0283), suggesting that SseC is conserved in human adapted serovars (*S.* Typhi and *S.* Paratyphi) and in serovars that can infect multiple hosts (*S.* Paratyphi and *S.* Typhimurium) (Fig. 4A and Table 3). Interestingly, in accordance with our observation, it is reported that the *sseC* of human adapted serovars shows a unique genetic polymorphism absent in other serovars [37]. D_N_ values of *sseD* between *S.* Typhi and *S.* Paratyphi and between *S.* Typhimurium and *S.* Enteritidis were zero indicating that SseD is identical in human adapted serovars and in serovars that can infect multiple hosts (Fig. 4B).

Human adapted serovars thus appear to have evolved different SseC and SseD that result in an altered translocon complex, which probably makes a more stable and effective contact with the phagosomal membrane of human cells enabling these serovars to survive and multiply inside human cells. Similarly, serovars that can infect multiple hosts may also have evolved a different translocon complex that enables contact with the phagosomal membranes of a wide range of hosts. Together, SseC and SseD, might help different serovars to recognize phagosomal membranes of their specific hosts in order to make a membrane pore and translocate effector proteins into host cells. In addition, differential evolution of *sseC* and *sseD* may also explain the differential survival and replication ability of human adapted serovars inside human and murine macrophages [22].

### Differential evolution of *sptP*

*sptP* encodes a 543 amino acid long secretory protein of SPI-1 encoded TTSS and has two functional domains: a tyrosine phosphatase domain (from position 300 to 543) and a GAP (GTPase activating protein) domain (from position 161 to 291) [38], [39]. SptP also has a SicP binding domain at its amino terminal (from position 35 to 139). SicP is a chaperone protein that binds to SptP and enables it to pass through the narrow channel of TTSS [40]. The cytoskeletal changes that promote the internalization of *Salmonella* are rapidly reversed by the GAP domain of SptP that targets Cdc42 and Rac1 of host cells [41]. SptP is also known to inhibit the mitogen-activated protein kinase pathway by inhibiting Raf activation through its tyrosine phosphatase activity [42], [43].

Like *sipD*, *sptP* is highly conserved in human adapted serovars, *S.* Typhi and *S.* Paratyphi, with a D_N_ value of 0.0048. *sptP* is also conserved among other serovars that are not adapted to humans (D_N_ = 0.0024 to 0.0032). The D_N_ values of *sptP* between human adapted serovars (*S.* Typhi and *S.* Paratyphi) and other serovars (*S.* Typhimurium, *S.* Choleraesuis, and *S.* Enteritidis) were high (0.0266 to 0.0295) suggesting that SptP of human adapted serovars is different from that of other serovars (Fig. 4C).

*S.* Typhimurium can trigger the migration of neutrophils across a monolayer of polarized colonic epithelial cells, whereas *S.* Typhi cannot elicit this response [44]. Furthermore, *S.* Typhi infection results in markedly reduced IL-8 production compared to infection with *S.* Typhimurium in the intestinal mucosa [45]. These reports suggest that unlike *S.* Typhimurium, *S.* Typhi down-regulates the host innate immune response in the intestinal mucosa, which probably helps S. Typhi disseminate into systemic circulation. NF-κB is a central regulator of the intestinal epithelial cell innate immune response induced by infection with enteroinvasive bacteria including *Salmonella* [46]. We speculate that SptP plays an important role in the differential innate immune response observed between *S.* Typhi and *S.* Typhimurium in the human intestine as SptP is known to inhibit mitogen-activated protein kinase pathway that activates NF-κB [42], [43].

### Differential evolution of *sseF*

**Figure 5:**
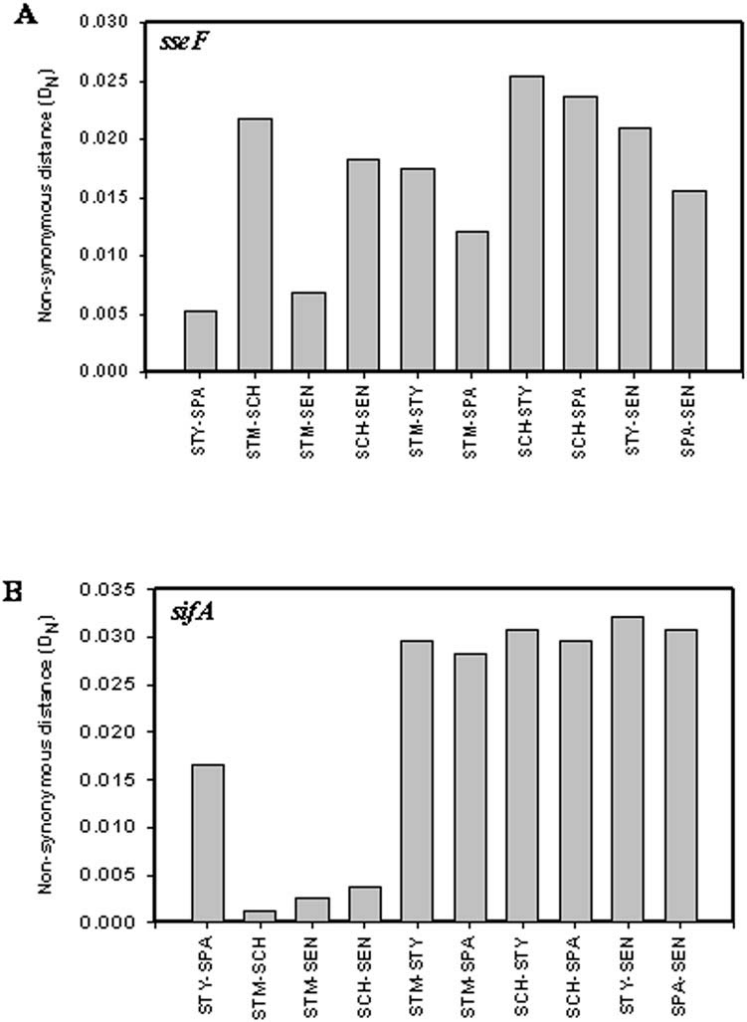
Differential evolution of *sseF* and *sifA*. (A) Non-synonymous distance profile of *sseF*. (B) Non-synonymous distance profile of *sifA*. STM- *S.* Typhimurium; STY- *S.* Typhi; SCH- *S.* Choleraesuis; SPA- *S.* Paratyphi; SEN- *S.* Enteritidis.

SseF is an effector protein secreted into the host cytoplasm through the SPI-2 encoded TTSS and is required to maintain the *Salmonella*-containing vacuole (SCV) in a juxtanuclear position by recruiting dynein [47]–[49]. D_N_ values of *sseF* between *S.* Typhi and *S.* Paratyphi and between *S.* Typhimurium and *S.* Enteritidis were small (0.005 and 0.0069, respectively) compared to other combinations of serovars (0.0121 to 0.0254) (Fig. 5A and Table 3). SseF is thus conserved in human adapted serovars and serovars that can infect multiple hosts. In support of our observations, *sseF,* like *sseC,* is shown to have a unique genetic polymorphism in human adapted serovars that is absent in other serovars [37]. Different serovars might have evolved different SseF in order to recruit dynein molecules of different hosts. *sseF* may thus be an important determinant of host specificity in human adapted serovars, acting at the intracellular phase of infection.

### Differential evolution of *sifA*

*sifA* encodes an effector protein that is translocated across the SCV membrane into the host cytoplasm through SPI-2 encoded TTSS and is located outside the SPI-2. SifA is necessary for the formation of *Salmonella*-induced filaments and maintains the integrity of SCV by down-regulating the recruitment of kinesin, which is necessary to maintain SCV in a juxtanuclear position [50]–[52].

The D_N_ values of *sifA* between human adapted serovars (*S.* Typhi and *S.* Paratyphi) and other serovars were high (0.0282 to 0.0321), suggesting that SifA of *S.* Typhimurium, *S.* Enteritidis and *S.* Choleraesuis are different from those of human adapted serovars (Fig. 5B). Alignment of predicted amino acid sequences of SifA from all these serovars revealed many favored and disfavored amino acid substitutions specific to human adapted serovars (Fig. S4; Materials and Methods S1). Human adapted serovars thus appear to have evolved a different SifA, which might help them maintain the integrity of the SCV in the human intracellular environment. The conserved N terminal motif, WEK(I/M)xxFF, implicated in intracellular targeting, was not altered. The last six amino-acids of SifA (331–336) are important for membrane anchoring and for its biological function [53]. Interestingly, the cysteine residue at position 331, which may serve as a recognition site for lipidation along with the other two cysteines (positions 333 and 334), was replaced by tyrosine in *S.* Typhi (Fig. S4). Lipidation is a post-translational modification and is important for membrane attachment and biological function of many proteins [54]. Post-translational modification of SifA in *S.* Typhi may thus be different from other serovars and may be important for the adaptation of *S.* typhi to humans.

### Conclusions

Using a molecular evolution and phylogenetics based approach, we have identified six genes that potentially underlie the host specificity of *S*. *enterica* serovars. Our study demonstrates that the translocon components of both SPI-1 (SipD) and SPI-2 (SseC and SseD) encoded TTSSs have evolved differentially among different serovars of *S*. *enterica*. The translocon components come in direct contact with the host cell membrane/phagosomal membrane which possibly necessitates their differential evolution for specific host adaptation. SseF and SifA, two effector molecules secreted through SPI-2 encoded TTSS, which interact (directly/indirectly) with two motor molecules, dynein and kinesin, whose recruitment influences the intracellular fate of *S*. *enterica*, are also differentially evolved. SptP, which can suppress the innate immune response at the intestinal level facilitating systemic spread of human adapted serovars in humans is also differentially evolved. Differentially evolved genes of SPI-1 encoded TTSS might act at the host cell invasion phase and those related to SPI-2 encoded TTSS might act at the intracellular phase of infection and together contribute to the host specificity of different serovars of *S*. *enterica* that are considered in our study. We recognize that our approach may not yield an exhaustive list of genes that underlie host specificity. Our approach, however, does provide a list of candidate genes that contribute substantially to host specificity. Our novel yet simple approach may be readily extended to other pathogens, such as *Mycobacteria*, whose species differ in their host specificity.

## Materials and Methods

### Genomic sequences

The genome sequences of *S.* Typhimurium LT2 (NC_003197), *S.* Typhi (Ty2) (NC_004631), *S.* Paratyphi A str. ATCC 9150 (NC_006511) and *S.* Choleraesuis str. SC-B67 (NC_006905) were taken from GenBank and the genome sequence of *S.* Enteritidis *PT4* (NCTC 13349) was taken from Sanger Institute webpage (http://www.sanger.ac.uk/Projects/Salmonella/). The genomic sequence of *S*. Enteritidis *PT4* (NCTC 13349) is not annotated. In order to obtain the sequences of SPI genes of this serovar we used NCBI BLAST and used *S.* Typhimurium LT2 (NC_003197) SPI gene sequences as query sequence [55].

### Non-synonymous distance calculation

Non-synonymous distance, D_N_, (the number of non-synonymous substitutions per non-synonymous site) was calculated using the DNA Sequence Polymorphism software DnaSP 4.0 (Version 4.10.9) [56]. Sequences with varied length were trimmed to a uniform length.

### Phylogenetic analysis

The sequences were aligned using ClustalW2 with default settings [57]. Phylip format of the output file of ClustalW2 was used to infer the phylogeny using TREE-PUZZLE 5.2 program [18]. Same program was used to construct the consensus tree of 95 genes belonging to SPI-1 to SPI-5. The outtree file was used to construct phylogenic trees using TreeView program [58]. To test for the significance of differences in likelihoods between trees, we used TREE-PUZZLE 5.2 implementation of the Shimodaira-Hasegawa (SH) test. This test was performed with 1000 resampling using RELL method and 5% significance level was used.

### Bacterial strains and growth conditions

*Salmonella enterica* serovar Typhi strain CT18 (Obtained from Institute of Microbial Technology, Chandigarh, India) and *Salmonella enterica* serovar Typhimurium 12023 (Gifted by Prof. Michael Hensel, Germany) were used in invasion experiments. Bacteria were routinely cultured in LB medium. The *sipD* deletion strains (Δ*sipD S.* Typhi and Δ*sipD S.* Typhimurium) were grown in medium containing kanamycin (50 µg/ml) and complemented strains carrying plasmids were grown in medium containing ampicillin (50 µg/ml).

### Construction of non-polar *sipD* null mutants (Δ*sipD*) of *S.* Typhi and *S.* Typhimurium

*sipD* gene was deleted using one-step deletion strategy [59]. *sipD* gene was replaced by kanamycin resistance marker from pKD4 using Lambda Red recombinase system. *sipD* null mutant was confirmed by colony PCR. Same set of primers were used for both *S.* Typhi and *S.* Typhimurium (Table S7).

### Complementation of Δ*sipD Salmonella*

The *sipD* gene from both *S.* Typhimurium and *S.* Typhi was amplified using primers (Table S7) having NcoI and SalI restriction enzyme sites. The resulting PCR amplified genes from both the serovars were introduced between the NcoI and SalI sites of the pTrc99aΔLacI plasmid to get pTrc-STM*sipD* and pTrc-STY*sipD*. Then pTrc-STM*sipD* and pTrc-STY*sipD* were electroporated into Δ*sipD S.* Typhi and Δ*sipD S.* Typhimurium to get respective complemented strains.

### Invasion assay

HeLa and Intestine 407 cells were used for the invasion assays. The cells were grown in antibiotic free Dulbecco's Modified Eagle's Medium (DMEM; Sigma) with 10% fetal calf serum (Sigma) at 37°C and 5% CO_2_. Cells were seeded at a density of 1.5×10^5^ cells per well in a 24-well plate. Bacteria were grown overnight in LB medium at 37°C and then they were subcultured in fresh LB medium at 1:33 ratio. The subcultures were then grown for 3 h after which the bacterial cells were washed in PBS and used to infect HeLa and Intestine 407 cells at a multiplicity of infection of 1:1. After infection, the plates were centrifuged at 1000 rpm for 5 min followed by 20 min incubation at 37°C and 5% CO_2_. The cells were then washed 5–6 times in warm PBS, followed by 30 min incubation in DMEM containing 100 µg/ml gentamicin (Sigma) to get rid of extracellular bacteria. After 30 min, the cells were again washed 3 times with warm PBS and lysed using PBS containing 0.1% TritonX-100 (Sigma), the lysate was plated on LB agar having specific antibiotic and the numbers of bacteria were enumerated after 12 h incubation. The invasion was calculated as the percentage of bacteria that entered as against the pre-inoculum for each strain. The infection was carried out in triplicate wells for each strain and the whole experiment was repeated thrice.

### RT-PCR

Bacterial RNA was extracted from log phase culture grown in LB using TRI Reagent (Sigma) and treated with RNase-free DNase (Fermantas) to digest the contaminant DNA. The DNA-free RNA was then reverse transcribed using reverse transcription system (Promega) using gene specific primer (*sipD* and *rpoD*) and amplified (35 cycles) by PCR. Primers used are presented in Table S7.

## Supporting Information

Table S1 Synonymous distance values of SPI-1 genes for all serovar combinations
(0.02 MB XLS)

Table S2 Synonymous distance values of SPI-2 genes for all serovar combinations
(0.02 MB XLS)

Table S3 Synonymous distance values of SPI-3 genes for all serovar combinations
(0.01 MB XLS)

Table S4 Synonymous distance values of SPI-4 genes for all serovar combinations
(0.01 MB XLS)

Table S5 Synonymous distance values of SPI-5 genes for all serovar combinations
(0.01 MB XLS)

Table S6 Genes belonging to SPI-1 to SPI-5 that are not included in this study
(0.04 MB DOC)

Table S7 Primers used in this study
(0.03 MB DOC)

Materials and Methods S1 (0.04 MB DOC)

Figure S1 Alignment of predicted amino acid sequences of SipD from different serovars of *Salmonella*. Positions showing amino acid changes are shaded. Arrow heads represent disfavored amino acid substitutions.
(7.05 MB TIF)

Figure S2 Predicted tertiary structures of SipD of (A) *S*. Typhimurium and (B) *S*. Typhi. These structures were obtained using Phyre software. These two structures differ at three regions indicated in white circles (A, B and C). These regions correspond to the amino acid residues 47 to 57(A), 268 to 282 (B) and 197 to 210 (C).
(5.43 MB TIF)

Figure S3 (A) Expression of *S*. Typhi SipD but not *S*. Typhimurium SipD enabled ΔsipD*S*. Typhi to enter Intestine 407 cells. (B) Expression of either *S*. Typhi SipD or *S*. Typhimurium SipD enabled ΔsipD *S*. Typhimurium to enter Intestine 407 cells. Graphs represent mean % entry. Error bars represent standard error. Student's ‘t’-test was used to calculate the P values. (C) Westernblot analysis of SipC expression in different strains of *Salmonella* (as indicated). STM-*S*. Typhimurium, STY-*S*. Typhi.
(1.64 MB TIF)

Figure S4 Alignment of amino acid sequences of SifA from different serovars of *S. enterica*. Positions showing amino acid changes are shaded. Arrow heads represent disfavored amino acid substitutions.
(4.91 MB TIF)

## References

[1] Reeves MW, Evins GM, Heiba AA, Plikaytis BD, Farmer JJ (1989). Clonal nature of Salmonella typhi and its genetic relatedness to other salmonellae as shown by multilocus enzyme electrophoresis, and proposal of Salmonella bongori comb. nov.. J Clin Microbiol.

[2] Baumler AJ, Tsolis RM, Ficht TA, Adams LG (1998). Evolution of host adaptation in Salmonella enterica.. Infect Immun.

[3] Uzzau S, Leori GS, Petruzzi V, Watson PR, Schianchi G (2001). Salmonella enterica serovar-host specificity does not correlate with the magnitude of intestinal invasion in sheep.. Infect Immun.

[4] Barrow PA, Huggins MB, Lovell MA (1994). Host specificity of Salmonella infection in chickens and mice is expressed in vivo primarily at the level of the reticuloendothelial system.. Infect Immun.

[5] Pascopella L, Raupach B, Ghori N, Monack D, Falkow S (1995). Host restriction phenotypes of Salmonella typhi and Salmonella gallinarum.. Infect Immun.

[6] Paulin SM, Watson PR, Benmore AR, Stevens MP, Jones PW (2002). Analysis of Salmonella enterica serotype-host specificity in calves: avirulence of S. enterica serotype gallinarum correlates with bacterial dissemination from mesenteric lymph nodes and persistence in vivo.. Infect Immun.

[7] Morgan E, Campbell JD, Rowe SC, Bispham J, Stevens MP (2004). Identification of host-specific colonization factors of Salmonella enterica serovar Typhimurium.. Mol Microbiol.

[8] Edwards RA, Olsen GJ, Maloy SR (2002). Comparative genomics of closely related salmonellae.. Trends Microbiol.

[9] Hansen-Wester I, Chakravortty D, Hensel M (2004). Functional transfer of Salmonella pathogenicity island 2 to Salmonella bongori and Escherichia coli.. Infect Immun.

[10] Groisman EA, Ochman H (1996). Pathogenicity islands: bacterial evolution in quantum leaps.. Cell.

[11] Mills DM, Bajaj V, Lee CA (1995). A 40 kb chromosomal fragment encoding Salmonella typhimurium invasion genes is absent from the corresponding region of the Escherichia coli K-12 chromosome.. Mol Microbiol.

[12] Collazo CM, Galan JE (1997). The invasion-associated type-III protein secretion system in Salmonella–a review.. Gene.

[13] Shea JE, Hensel M, Gleeson C, Holden DW (1996). Identification of a virulence locus encoding a second type III secretion system in Salmonella typhimurium.. Proc Natl Acad Sci U S A.

[14] Hensel M (2000). Salmonella pathogenicity island 2.. Mol Microbiol.

[15] Blanc-Potard AB, Groisman EA (1997). The Salmonella selC locus contains a pathogenicity island mediating intramacrophage survival.. Embo J.

[16] Gerlach RG, Jackel D, Stecher B, Wagner C, Lupas A (2007). Salmonella Pathogenicity Island 4 encodes a giant non-fimbrial adhesin and the cognate type 1 secretion system.. Cell Microbiol.

[17] Wood MW, Jones MA, Watson PR, Hedges S, Wallis TS (1998). Identification of a pathogenicity island required for Salmonella enteropathogenicity.. Mol Microbiol.

[18] Schmidt HA, Strimmer K, Vingron M, von Haeseler A (2002). TREE-PUZZLE: maximum likelihood phylogenetic analysis using quartets and parallel computing.. Bioinformatics.

[19] Shimodaira H, Hasegawa M (1999). Multiple Comparisons of Log-Likelihoods with Applications to Phylogenetic Inference.. Mol Biol Evol.

[20] Galan JE (2001). Salmonella interactions with host cells: type III secretion at work.. Annu Rev Cell Dev Biol.

[21] Darwin KH, Miller VL (1999). Molecular basis of the interaction of Salmonella with the intestinal mucosa.. Clin Microbiol Rev.

[22] Schwan WR, Huang XZ, Hu L, Kopecko DJ (2000). Differential bacterial survival, replication, and apoptosis-inducing ability of Salmonella serovars within human and murine macrophages.. Infect Immun.

[23] Kaniga K, Trollinger D, Galan JE (1995). Identification of two targets of the type III protein secretion system encoded by the inv and spa loci of Salmonella typhimurium that have homology to the Shigella IpaD and IpaA proteins.. J Bacteriol.

[24] Collazo CM, Galan JE (1997). The invasion-associated type III system of Salmonella typhimurium directs the translocation of Sip proteins into the host cell.. Mol Microbiol.

[25] Espina M, Olive AJ, Kenjale R, Moore DS, Ausar SF (2006). IpaD localizes to the tip of the type III secretion system needle of Shigella flexneri.. Infect Immun.

[26] Mueller CA, Broz P, Muller SA, Ringler P, Erne-Brand F (2005). The V-antigen of Yersinia forms a distinct structure at the tip of injectisome needles.. Science.

[27] Cornelis GR (2006). The type III secretion injectisome.. Nat Rev Microbiol.

[28] Picking WL, Nishioka H, Hearn PD, Baxter MA, Harrington AT (2005). IpaD of Shigella flexneri is independently required for regulation of Ipa protein secretion and efficient insertion of IpaB and IpaC into host membranes.. Infect Immun.

[29] Zhang S, Kingsley RA, Santos RL, Andrews-Polymenis H, Raffatellu M (2003). Molecular pathogenesis of Salmonella enterica serotype typhimurium-induced diarrhea.. Infect Immun.

[30] Bhan MK, Bahl R, Bhatnagar S (2005). Typhoid and paratyphoid fever.. Lancet.

[31] Weinstein DL, O'Neill BL, Hone DM, Metcalf ES (1998). Differential early interactions between Salmonella enterica serovar Typhi and two other pathogenic Salmonella serovars with intestinal epithelial cells.. Infect Immun.

[32] Pier GB, Grout M, Zaidi T, Meluleni G, Mueschenborn SS (1998). Salmonella typhi uses CFTR to enter intestinal epithelial cells.. Nature.

[33] Lawley TD, Chan K, Thompson LJ, Kim CC, Govoni GR (2006). Genome-wide screen for Salmonella genes required for long-term systemic infection of the mouse.. PLoS Pathog.

[34] Nikolaus T, Deiwick J, Rappl C, Freeman JA, Schroder W (2001). SseBCD proteins are secreted by the type III secretion system of Salmonella pathogenicity island 2 and function as a translocon.. J Bacteriol.

[35] Klein JR, Jones BD (2001). Salmonella pathogenicity island 2-encoded proteins SseC and SseD are essential for virulence and are substrates of the type III secretion system.. Infect Immun.

[36] Hensel M, Shea JE, Waterman SR, Mundy R, Nikolaus T (1998). Genes encoding putative effector proteins of the type III secretion system of Salmonella pathogenicity island 2 are required for bacterial virulence and proliferation in macrophages.. Mol Microbiol.

[37] Tracz DM, Tabor H, Jerome M, Ng LK, Gilmour MW (2006). Genetic determinants and polymorphisms specific for human-adapted serovars of Salmonella enterica that cause enteric fever.. J Clin Microbiol.

[38] Kaniga K, Uralil J, Bliska JB, Galan JE (1996). A secreted protein tyrosine phosphatase with modular effector domains in the bacterial pathogen Salmonella typhimurium.. Mol Microbiol.

[39] Stebbins CE, Galan JE (2001). Maintenance of an unfolded polypeptide by a cognate chaperone in bacterial type III secretion.. Nature.

[40] Fu Y, Galan JE (1998). Identification of a specific chaperone for SptP, a substrate of the centisome 63 type III secretion system of Salmonella typhimurium.. J Bacteriol.

[41] Fu Y, Galan JE (1999). A salmonella protein antagonizes Rac-1 and Cdc42 to mediate host-cell recovery after bacterial invasion.. Nature.

[42] Lin SL, Le TX, Cowen DS (2003). SptP, a Salmonella typhimurium type III-secreted protein, inhibits the mitogen-activated protein kinase pathway by inhibiting Raf activation.. Cell Microbiol.

[43] Murli S, Watson RO, Galan JE (2001). Role of tyrosine kinases and the tyrosine phosphatase SptP in the interaction of Salmonella with host cells.. Cell Microbiol.

[44] McCormick BA, Miller SI, Carnes D, Madara JL (1995). Transepithelial signaling to neutrophils by salmonellae: a novel virulence mechanism for gastroenteritis.. Infect Immun.

[45] Raffatellu M, Chessa D, Wilson RP, Dusold R, Rubino S (2005). The Vi capsular antigen of Salmonella enterica serotype Typhi reduces Toll-like receptor-dependent interleukin-8 expression in the intestinal mucosa.. Infect Immun.

[46] Elewaut D, DiDonato JA, Kim JM, Truong F, Eckmann L (1999). NF-kappa B is a central regulator of the intestinal epithelial cell innate immune response induced by infection with enteroinvasive bacteria.. J Immunol.

[47] Hansen-Wester I, Stecher B, Hensel M (2002). Type III secretion of Salmonella enterica serovar Typhimurium translocated effectors and SseFG.. Infect Immun.

[48] Deiwick J, Salcedo SP, Boucrot E, Gilliland SM, Henry T (2006). The translocated Salmonella effector proteins SseF and SseG interact and are required to establish an intracellular replication niche.. Infect Immun.

[49] Abrahams GL, Muller P, Hensel M (2006). Functional dissection of SseF, a type III effector protein involved in positioning the salmonella-containing vacuole.. Traffic.

[50] Stein MA, Leung KY, Zwick M, Garcia-del Portillo F, Finlay BB (1996). Identification of a Salmonella virulence gene required for formation of filamentous structures containing lysosomal membrane glycoproteins within epithelial cells.. Mol Microbiol.

[51] Beuzon CR, Meresse S, Unsworth KE, Ruiz-Albert J, Garvis S (2000). Salmonella maintains the integrity of its intracellular vacuole through the action of SifA.. Embo J.

[52] Boucrot E, Henry T, Borg JP, Gorvel JP, Meresse S (2005). The intracellular fate of Salmonella depends on the recruitment of kinesin.. Science.

[53] Boucrot E, Beuzon CR, Holden DW, Gorvel JP, Meresse S (2003). Salmonella typhimurium SifA effector protein requires its membrane-anchoring C-terminal hexapeptide for its biological function.. J Biol Chem.

[54] Casey PJ (1995). Protein lipidation in cell signaling.. Science.

[55] Altschul SF, Gish W, Miller W, Myers EW, Lipman DJ (1990). Basic local alignment search tool.. J Mol Biol.

[56] Rozas J, Sanchez-DelBarrio JC, Messeguer X, Rozas R (2003). DnaSP, DNA polymorphism analyses by the coalescent and other methods.. Bioinformatics.

[57] Larkin MA, Blackshields G, Brown NP, Chenna R, McGettigan PA (2007). Clustal W and Clustal X version 2.0.. Bioinformatics.

[58] Page RD (1996). TreeView: an application to display phylogenetic trees on personal computers.. Comput Appl Biosci.

[59] Datsenko KA, Wanner BL (2000). One-step inactivation of chromosomal genes in Escherichia coli K-12 using PCR products.. Proc Natl Acad Sci U S A.

