# Supplementary material for: Differentially Evolved Genes of Salmonella Pathogenicity Islands: Insights into the Mechanism of Host Specificity in Salmonella
Source: PLoS One. 2008 Dec 3;3(12):e3829. doi: 10.1371/journal.pone.0003829 (PMC2585142; doi:10.1371/journal.pone.0003829)
Supplement: Table S6 — Genes belonging to SPI-1 to SPI-5 that are not included in this study (0.04 MB DOC) [file pone.0003829.s006.doc]

**Table S6. Genes belonging to SPI-1 to SPI-5 that are not included in this study***

| **Pathogenecity Island** | **Gene^#^** |
| --- | --- |
| SPI-1 | *avrA,* |
|  | STM2901 |
|  | STM2902 |
|  | STM2903 |
| Encode effector proteins  secreted via SPI-2 encoded  TTSS | *sspH1* |
|  | *sspH2* |
|  | *sifB* |
|  | *sseJ* |
|  | *sseI* |
|  | *slrP* |
|  | *sopD2* |
| SPI-3 | *marT* |
|  | *misL,* |
|  | *cigR* |
|  | *rhuM* |
| SPI-4 | STM4261 |
| SPI-5 | *pipD* |

* These genes were not suitable for our analysis as they were either pseudogenes or

absent in some of the serovars considered in our study.
