# Supplementary material for: Differentially Evolved Genes of Salmonella Pathogenicity Islands: Insights into the Mechanism of Host Specificity in Salmonella
Source: PLoS One. 2008 Dec 3;3(12):e3829. doi: 10.1371/journal.pone.0003829 (PMC2585142; doi:10.1371/journal.pone.0003829)
Supplement: Table S7 — Primers used in this study (0.03 MB DOC) [file pone.0003829.s007.doc]

| Deletion of *sipD* | Forward: tcttacacttgtaaccattattaatatcctcttctggtgtaggctggagctgcttcg  Reverse: atttaatcgccctcctgatggcgaactggggatattcatatgaatatcctcctta |
| --- | --- |
| Confirmation of *sipD*  deletion | Forward: gcaagattcgtggcctgcgt  Reverse: cgcactcgctgctatcgcag |
| Cloning of *sipD* | Forawrd: atgcccatgggacttaatattcaaaattattccgct  Reverse: atgcgtcgacttatccttgcaggaagcttttggc |
| RT-PCR of *sipD* | Forward: attcactgag ccactgtctc  Reverse: gctagatgttacctcactc |
| RT-PCR of *rpoD* | Forward: gtggcttgcaattccttgat  Reverse: agcatctggcgagaaatacg |

**Table S7. Primers* used in this study**

* Primers are given in 5' to 3' direction
