# Supplementary material for: Differentially Evolved Genes of Salmonella Pathogenicity Islands: Insights into the Mechanism of Host Specificity in Salmonella
Source: PLoS One. 2008 Dec 3;3(12):e3829. doi: 10.1371/journal.pone.0003829 (PMC2585142; doi:10.1371/journal.pone.0003829)
Supplement: Materials and Methods S1 — (0.04 MB DOC) [file pone.0003829.s008.doc]

**Materials and Methods S1**

**Protein sequence analysis**

The multiple sequence alignment of the protein sequences was performed by the BLOSUM 62 substitution matrix of MULTALIN software [1]. The protein sequences were arranged individually with postulated gaps so that similar residues were juxtaposed. Favored and disfavored amino acid substitutions were identified as described previously [2].

**Prediction of tertiary structure**

Amino acid sequences of SipD of *S*. Typhimurium (LT2) and *S*. Typhi (TY2) were retrieved from NCBI. These sequences were used to predict 3D structure using Phyre software (<http://www.sbg.bio.ic.ac.uk/phyre/>) [3].

**Westernblot analysis**

Bacteria were cultured in 5 ml LB containing 0.3 M NaCl in a tube with a tightly closed lid For SPI1 induction [4]. Cultures were incubated for 12 h at 37°C with shaking. After 12 h, equal no. of bacteria (inferred from the optical density) were pelleted and the pellet containing bacteria were resuspended in SDS–PAGE sample buffer. Equal amount pf protein was separated on 10% SDS–PAGE gel after boiling for 5 min. Protein was transferred onto nitrocellulose membranes (Millipore). SipC was detected using anti-SipC antibody raised in rabbit [5] (a kind gift from Prof. Michael Hensel) and HRP conjugated anti rabbit IgG antibody (Bangalore Genei). Immune complexes were detected using an enhanced chemiluminescence reagent (PerkinElmer).

**References for Supporting Information**

1. Corpet F (1988) Multiple sequence alignment with hierarchical clustering. Nucleic Acids Res 16: 10881-10890.

2. Betts MJ, Russell RB (2003) Amino acid properties and consequences of subsitutions.

In: Barnes MR, Gray IC, editor. Bioinformatics for Geneticists Wiley.

3. Bennett-Lovsey RM, Herbert AD, Sternberg MJ, Kelley LA (2008) Exploring the

extremes of sequence/structure space with ensemble fold recognition in the program

Phyre. Proteins 70: 611-625.

4. Sittka A, Pfeiffer V, Tedin K, Vogel J (2007) The RNA chaperone Hfq is essential for

the virulence of Salmonella typhimurium. Molecular Microbiology 63: 193–217.

5. Deiwick J, Nikolaus T, Shea JE, Gleeson C, Holden DW, et al. (1998) Mutations in

Salmonella pathogenicity island 2 (SPI2) genes affecting transcription of SPI1 genes and

resistance to antimicrobial agents. J Bacteriol 180: 4775-4780.
