# Supplementary figures and images for: Differentially Evolved Genes of Salmonella Pathogenicity Islands: Insights into the Mechanism of Host Specificity in Salmonella
Source: PLoS One. 2008 Dec 3;3(12):e3829. doi: 10.1371/journal.pone.0003829 (PMC2585142; doi:10.1371/journal.pone.0003829)

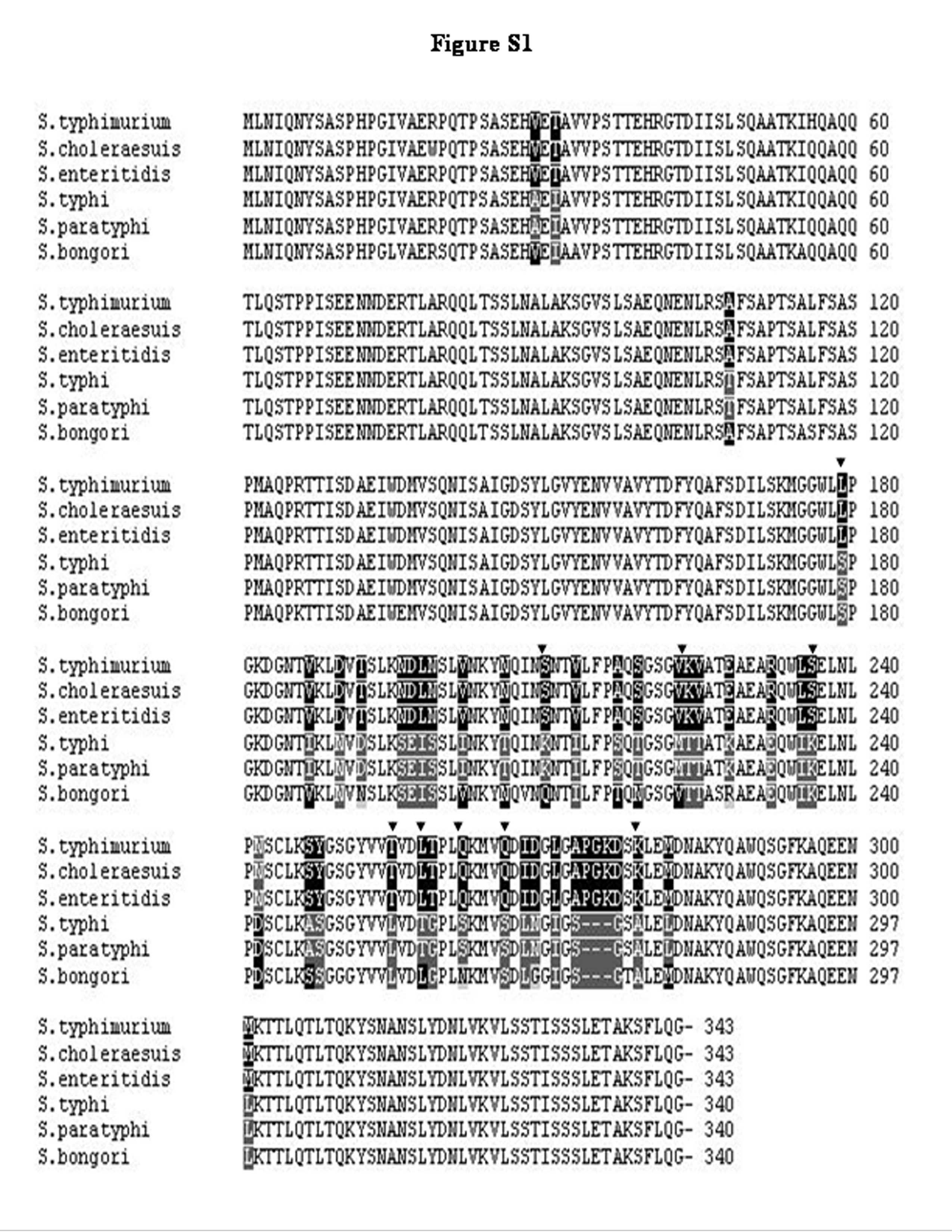

Supplement: Figure S1 — Alignment of predicted amino acid sequences of SipD from different serovars of Salmonella. Positions showing amino acid changes are shaded. Arrow heads represent disfavored amino acid substitutions. (7.05 MB TIF) [file pone.0003829.s009.tif]

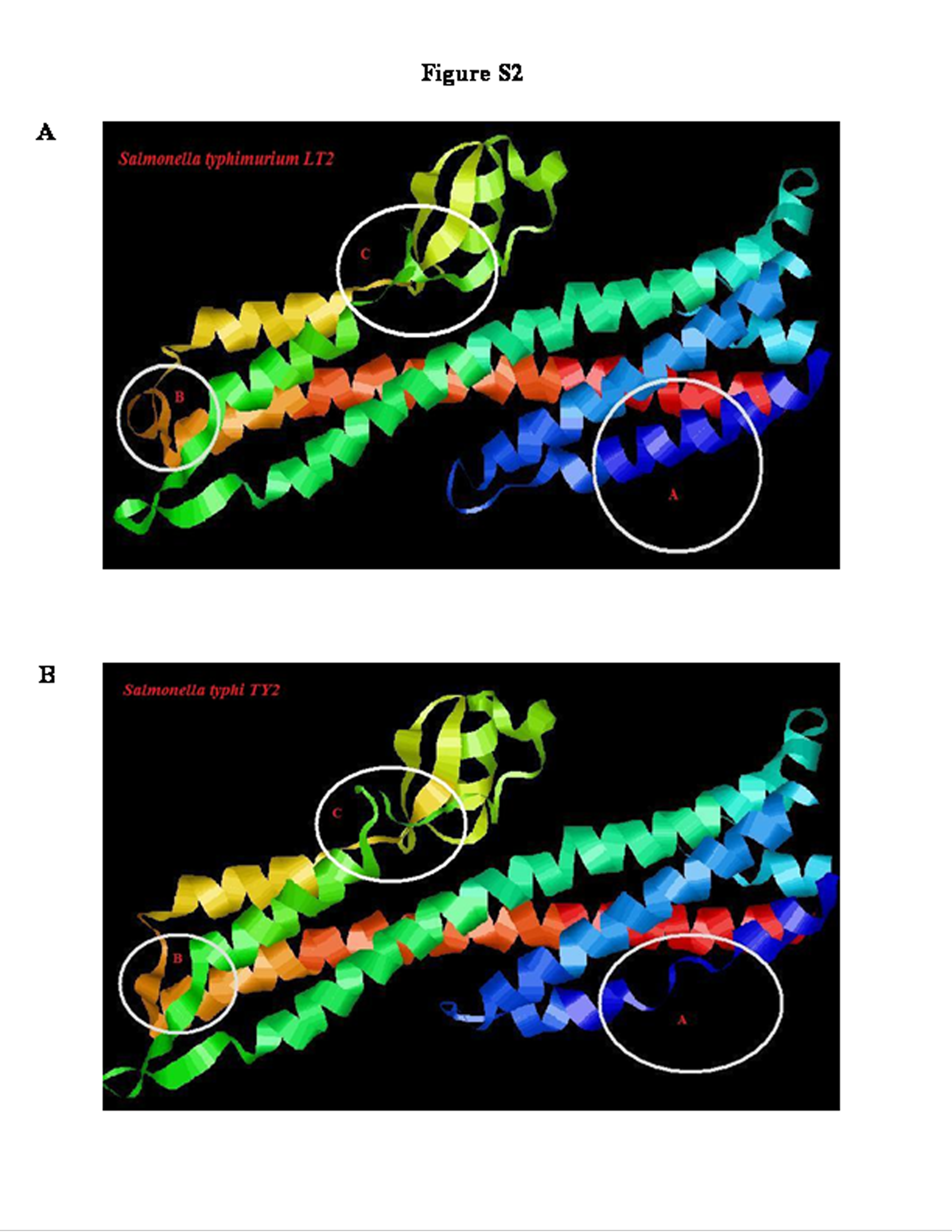

Supplement: Figure S2 — Predicted tertiary structures of SipD of (A) S. Typhimurium and (B) S. Typhi. These structures were obtained using Phyre software. These two structures differ at three regions indicated in white circles (A, B and C). These regions correspond to the amino acid residues 47 to 57(A), 268 to 282 (B) and 197 to 210 (C). (5.43 MB TIF) [file pone.0003829.s010.tif]

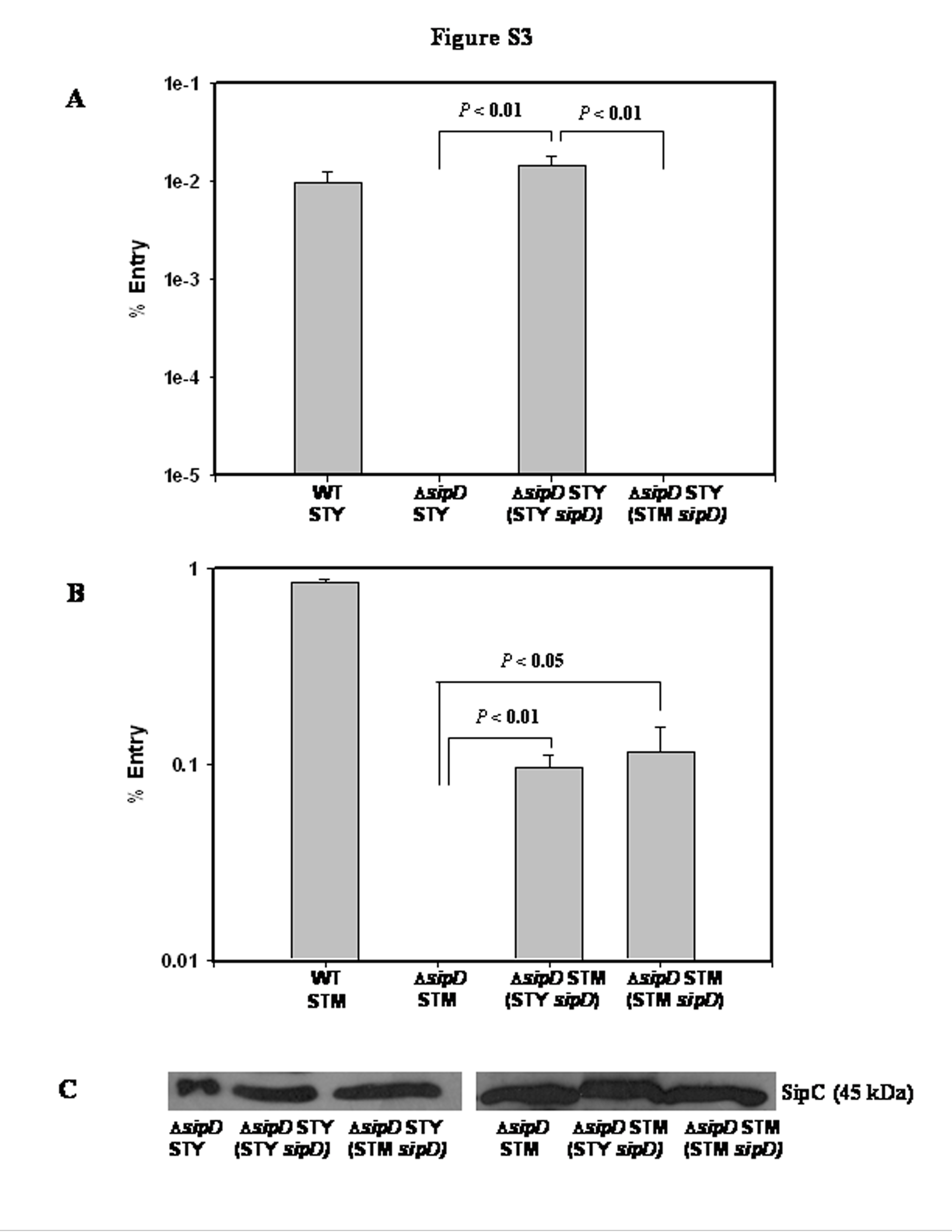

Supplement: Figure S3 — (A) Expression of S. Typhi SipD but not S. Typhimurium SipD enabled ΔsipDS. Typhi to enter Intestine 407 cells. (B) Expression of either S. Typhi SipD or S. Typhimurium SipD enabled ΔsipD S. Typhimurium to enter Intestine 407 cells. Graphs represent mean % entry. Error bars represent standard error. Student's ‘t’-test was used to calculate the P values. (C) Westernblot analysis of SipC expression in different strains of Salmonella (as indicated). STM-S. Typhimurium, STY-S. Typhi. (1.64 MB TIF) [file pone.0003829.s011.tif]

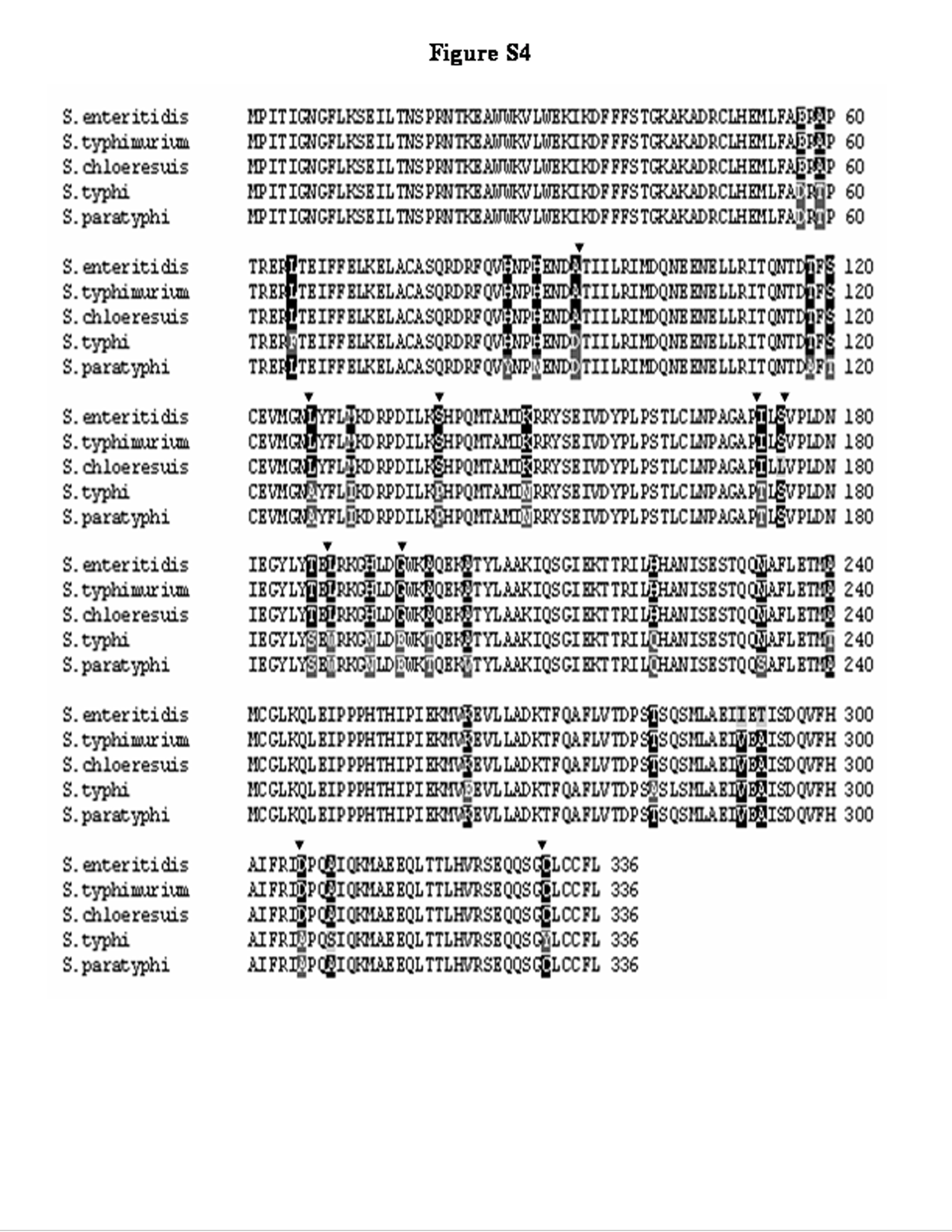

Supplement: Figure S4 — Alignment of amino acid sequences of SifA from different serovars of S. enterica. Positions showing amino acid changes are shaded. Arrow heads represent disfavored amino acid substitutions. (4.91 MB TIF) [file pone.0003829.s012.tif]
